# Marine Fungal Metabolites as Potential Antidiabetic Agents: A Comprehensive Review of Their Structures and Enzyme Inhibitory Activities

**DOI:** 10.3390/md23040142

**Published:** 2025-03-26

**Authors:** Zimin Wang, Meirong Zhao, Yunxia Yu, Fandong Kong, Nanxin Lin, Qi Wang

**Affiliations:** 1 Department of Pediatric intensive Care Medicine, Hainan Women and Children’s Medical Center, Haikou 570100, China; wzm55802023@163.com; 2 College of Food and Pharmaceutical Engineering, Guangxi Vocational University of Agriculture, Nanning 530006, China; zhaomeirong124@163.com; 3 Key Laboratory of Chemistry and Engineering of Forest Products, State Ethnic Affairs Commission, Guangxi Key Laboratory of Polysaccharide Materials and Modification, School of of Marine Science and Biotechnology, Guangxi Minzu University, Nanning 530006, China; yuyunxia2023@163.com

**Keywords:** diabetes mellitus, type 2 diabetes, marine fungal strains, bioactive compounds, diabetes-related enzymes, antidiabetic agents

## Abstract

Diabetes mellitus has emerged as a global public health crisis, with Type 2 diabetes (T2D) constituting over 90% of cases. Current treatments are palliative, primarily focusing on blood glucose modulation. This review systematically evaluates 181 bioactive compounds isolated from 66 marine fungal strains for their inhibitory activities against key diabetes-related enzymes, including α-glucosidase, protein tyrosine phosphatase 1B (PTP1B), dipeptidyl peptidase-4 (DPP-4), glycogen synthase kinase-3β (GSK-3β), and fatty acid-binding protein 4 (FABP4). These compounds, categorized into polyketides, alkaloids, terpenoids, and lignans, exhibit multitarget engagement and nanomolar-to-micromolar potency. The review highlights the potential of marine fungal metabolites as novel antidiabetic agents, emphasizing their structural novelty and diverse mechanisms of action. Future research should focus on overcoming challenges related to yield and extraction, leveraging advanced technologies such as genetic engineering and synthetic biology to enhance drug development.

## 1. Introduction

Diabetes mellitus, a chronic metabolic disorder characterized by persistent hyperglycemia, has emerged as a global public health crisis. The escalating prevalence of diabetes—driven by widespread adoption of high-caloric diets, sedentary behaviors, and demographic aging—not only predisposes individuals to life-threatening complications (e.g., cardiovascular diseases, nephropathy, and retinopathy) but also imposes substantial socioeconomic burdens on healthcare systems worldwide [1]. According to the ninth edition of the International Diabetes Federation (IDF) Diabetes Atlas (2019), diabetes now ranks as the most prevalent non-communicable disease (NCD), affecting approximately 463 million adults globally [2]. Pathophysiologically, this disorder stems from metabolic dysregulation caused by absolute or relative insulin deficiency coupled with aberrant glucagon activity [3]. Despite advances in glycemic management strategies, current therapies remain palliative rather than curative, focusing primarily on delaying complications through blood glucose modulation. Clinically, diabetes is classified into insulin-dependent Type 1 diabetes (T1D) and non-insulin-dependent Type 2 diabetes (T2D), with T2D constituting >90% of total cases [4].

First-line pharmacological interventions for T2D include insulin secretagogues (e.g., sulfonylureas), biguanides (e.g., metformin), α-glucosidase inhibitors (e.g., acarbose), and insulin sensitizers (e.g., thiazolidinediones) [5]. While these agents demonstrate initial therapeutic efficacy, their long-term utility is hampered by progressive β-cell dysfunction, acquired drug resistance, and adverse effects ranging from hypoglycemia to cardiovascular risks [6]. Consequently, the development of novel antidiabetic agents with improved safety profiles and mechanisms circumventing these limitations represents a critical unmet need in diabetes research. The pathogenesis of T2D involves intricate interactions between genetic predisposition and environmental triggers, manifesting as the dysregulation of key metabolic enzymes and signaling pathways. Central to this complexity are five pivotal targets: (1) α-glucosidase, which modulates postprandial hyperglycemia via carbohydrate digestion; (2) protein tyrosine phosphatase 1B (PTP1B), an insulin receptor signaling antagonist; (3) dipeptidyl peptidase-4 (DPP-4), responsible for incretin hormone degradation; (4) glycogen synthase kinase-3β (GSK-3β), a negative regulator of glycogen synthesis; and (5) fatty acid-binding protein 4 (FABP4), a mediator of lipid-induced insulin resistance and inflammation [7]. Targeting these enzymes offers dual advantages, elucidating molecular drivers of diabetes while enabling the rational design of multitarget therapeutics.

Natural products remain indispensable in drug discovery, with >60% of FDA-approved small-molecule drugs originating from natural scaffolds [8]. Among biological sources, fungi—particularly marine-derived strains—represent an underexplored reservoir of bioactive metabolites. Marine fungi, evolutionarily adapted to extreme environments (e.g., high salinity, low temperature, and oligotrophic conditions), exhibit unique biosynthetic pathways yielding structurally novel compounds with pronounced pharmacological potential [9]. Notably, marine fungal metabolites spanning polyketides, alkaloids, and terpenoids have demonstrated inhibitory effects against diabetes-related targets, positioning them as promising candidates for antidiabetic drug development [10].

This review systematically evaluates 181 bioactive compounds isolated from 66 marine fungal strains, focusing on their inhibitory activities against α-glucosidase (IC_50_: 0.8–58.3 μM), PTP1B (IC_50_: 1.2–32.7 μM), DPP-4 (IC50: 3.4–89.1 μM), GSK-3β (IC_50_: 0.5–47.6 μM), and FABP4 (IC_50_: 2.1–75.4 μM). Representative molecules include xylarianaphthol-1 (dual α-glucosidase/PTP1B inhibition, IC_50_ = 2.3/4.7 μM) and aspergoterpenin C (potent GSK-3β inhibitor, IC_50_ = 0.5 μM) [11]. Their structural novelty, multitarget engagement, and nanomolar-to-micromolar potency underscore their potential as lead compounds for next-generation antidiabetic therapeutics.

## 2. Compounds

**Figure 1:**
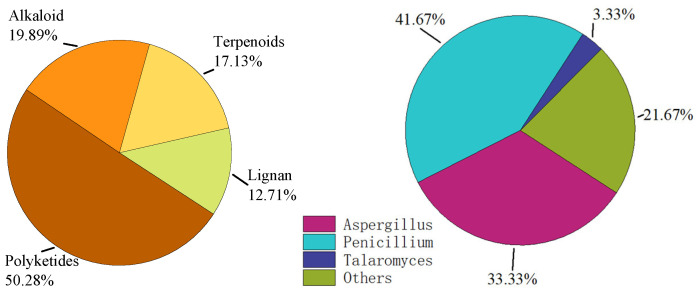
Proportions of compounds with different structures and fungal sources.

In the following sections, we will discuss these compounds based on their different structures and the activity of these compounds on diabetes-related enzymes. We divided these 181 compounds into four categories: polyketones, alkaloids, terpenes, and lignans, and their proportions are shown in Figure 1 below. These compounds were isolated from 66 strains of fungi sourced from diverse marine environments. The majority of these fungi belonged to the genera Penicillium and Aspergillus, while other fungi constituted merely 26.23%, representing a relatively small proportion. The detailed statistics are presented in Figure 1 below.

### 2.1. Polyketides

Polyketides, a class of natural products isolated from marine fungi, feature an extensive array of diverse structures synthesized via distinct biosynthetic pathways [12]. In the realm of diabetes drug research and development, these compounds have emerged as highly promising candidates. Their unique chemical architectures endow them with specific biological activities, positioning polyketides as strong contenders to serve as novel lead compounds for diabetes treatment. This holds the promise of introducing innovative therapeutic solutions to address the challenges posed by diabetes.

Five known compounds, emodin (**1**), geodin hydrate (**2**), methyl dichloroasterrate (**3**), monomethylosoic acid (**4**), and epicoccolide B (**5**), were isolated from the marine-derived *Aspergillus flavipes* HN4-13 collected from a Lianyungang coastal sediment sample. Compounds **1** and **4** showed noncompetitive α-glucosidase inhibition, with K_i_/IC_50_ values of 0.79/19 μM and 2.8/90 μM, respectively. Compounds **2**, **3**, and **5** exhibited mixed α-glucosidase inhibition, with K_i_/IC_50_ values of (6.3, 5.5)/55 μM, (1.4, 0.60)/9.9 μM, and (2.5, 7.2)/33 μM, respectively, compared to acarbose (IC_50_ 101 μM) and 1-deoxynojirimycin (IC_50_ 79 μM) [13]. Two new dibenzo-α-pyrone derivatives, alternolides B and C (**6**–**7**), and the known altenuisol alternariol 1′-hydroxy-9-methyl ether (**8**), were isolated from the marine-derived *Alternaria alternata* LW37 via the OSMAC strategy. Compounds **6**, **7**, and **8** inhibited α-glucosidase, with IC_50_ values of 725.85 ± 4.75 μM, 451.25 ± 6.95 μM, and 6.27 ± 0.68 μM, respectively. Molecular docking analysis indicated that compounds **6**–**8** formed three, four, and six hydrogen bonds, respectively, with α-glycosidase, which closely correlated with their experimentally determined bioactivity potencies [14].

The fungal strain *Penicillium thomii* YPGA3, isolated from Yap Trench sediments, yielded three new chromone derivatives, penithochromones R−T (**9**–**11**), along with two known compounds—penithochromone C (**12**) and penithochromone A (**13**). Compounds **9**–**13** significantly inhibited α-glucosidase, with IC_50_ values ranging from 268 to 1017 µM, outperforming the positive control acarbose (1.3 mmol) [15]. A new phenolic compound, epicocconigrone C (**14**), along with five known phenolic compounds, epicocconigrone A (**15**), 2-(10-formyl-11,13-dihydroxy-12-methoxy-14-methyl)-6,7-dihydroxy-5-methyl-4-benzofurancarboxaldehyde (**16**), epicoccolide B (**17**), eleganketal A (**18**), and 2,3,4-trihydroxy-6(hydroxymethyl)-5-methylbenzyl-alcohol (**19**), were isolated from *Aspergillus insulicola* from deep-sea sediment. Compounds **14**–**19** potently inhibited α-glucosidase, with IC_50_ values ranging from 17.04 to 292.47 µM, better than acarbose (IC_50_ 822.97 µM). The number of hydroxyl groups in polyhydroxyphenolic compounds is crucial for α-glucosidase inhibitory activity, as reflected in the low IC_50_ values of compounds **15** and **17**, while structures with fewer hydroxyl groups (compounds **14** and **16**) exhibit minimal activity [16].

Four new polyketides—5-((*R*,1*Z*,3*E*)-6-hydroxy-1,3-heptadien-1-yl)-1,3-benzenediol (**20**), 4-carboxy-5-((*R*,1*Z*,3*E*)-6-hydroxy-1,3-heptadien-1-yl)-1,3-benzenediol (**21**), 4-carboxy-5-((1*Z*,3*E*)-1,3-heptadien-1-yl)-1,3-benzenediol (**22**), 5-((1*Z*,3*E*)-4-carboxy-1,3-butadienyl-1-yl)-1,3-benzenediol (**23**)—and three known compounds—penialidin A (**24**), 3,4-dihydroxybenzeneacetic acid (**25**), and ɛ-caprolactone derivative (**26**)—were isolated from *Penicillium* sp. TW58-16. Compounds **20**–**26** had strong α-glucosidase inhibitory effects, with inhibition rates of 73.2%, 55.6%, 74.4%, 32.0%, 36.9%, 88.0%, and 91.1% at 400 μM, respectively, which were comparable to or higher than that of acarbose [17].

One new *p*-terphenyl derivative, asperterphenylcins B (**27**), along with the previously described terphenyllin (**28**) and 3″-hydroxyterphenyllin (**29**), was obtained from the solid-rice culture of marine-derived *Aspergillus candidus* HM5-4 isolated from South China Sea sponges. Compounds **27**–**29** potently inhibited α-glucosidase, with IC_50_ values of 1.26 ± 0.19 μM, 2.16 ± 0.44 μM, and 13.22 ± 0.55 μM, respectively [18]. Three new thioester-containing benzoate derivatives, Eurothiocin C (**30**), Eurothiocin F (**31**), and Eurothiocin G (**32**), were isolated from the deep-sea fungus *Talaromyces indigoticus* FS688. Compound **30** showed potent α-glucosidase inhibitory activity, with an IC_50_ value of 5.4 μM, while compounds **31** and **32** had moderate inhibitory effects, with IC_50_ values of 33.6 μM and 72.1 μM, respectively [19]. Two pairs of novel salicylaldehyde derivative enantiomers, euroticins G (**33**–**34**) and euroticins H (**35**–**36**), along with the known compound eurotirumin (**37**), were isolated and characterized from the marine-derived fungus *Eurotium* sp. SCSIO F452. Compounds **33** to **37** inhibited α-glucosidase, with IC_50_ values ranging from 16.31 to 79.71 μM [20]. From the marine-derived fungus *Meira* sp. 1210CH-42, a new catecholic compound, meirols A (**38**), and a known analog, argovin (**39**), were isolated. Both **38** and **39** inhibited α-glucosidase, with IC_50_ values of 199.70 μM and 184.50 μM, respectively (acarbose, IC_50_ = 301.93 μM) [21]. A new depsidone, botryorhodine H (**40**), together with two known analogs, botryorhodine C (**41**) and botryorhodines D (**42**), was obtained from the mangrove endophytic fungus *Trichoderma* sp. 307 through co-culturing with *Acinetobacter johnsonii* B2. Compounds **40** to **42** inhibited α-glucosidase, with IC_50_ values ranging from 8.1 to 11.2 μM [22]. A new chromone, 7-hydroxy-5-methoxy-2,3-dimethylchromone (**43**), and two known metabolites, 2,3-dihydro-5-methoxy-2-methylchromen-4-one (**44**) and helicascolides A (**45**), were isolated from the mangrove-derived fungus *Daldinia eschscholtzii* HJ004. Compounds **43** to **45** inhibited α-glucosidase, with IC_50_ values of 13 μM, 15 μM, and 16 μM, respectively [23]. Epicoccolide B (**46**), a known compound, was isolated from the mangrove fungus *Mycosphaerella* sp. SYSU-DZG01 and inhibited α-glucosidase, with an IC_50_ value of 26.7 μM [24]. Chemical investigation of the endophytic fungus *Aspergillus* sp. 16-5B, cultured on Czapek’s medium, led to the isolation of two new metabolites, aspergifuranone (**47**) and isocoumarin derivative 3,4-Dihydro-4,6,8-trihydroxy-3-methoxy-3,7-dimethyl-1*H*-2-benzopyran-1-one (**48**), along with the known pestaphthalide A (**49**). Compound **47** showed significant α-glucosidase inhibitory activity, with an IC_50_ value of 9.05 ± 0.60 μM. Compounds **48** and **49** had moderate inhibitory activities, with IC_50_ values of 90.4 μM and 96.6 μM, respectively [25].

Three biosynthetically related known analogs, penicidone C (**50**), penicillide (**51**), and Sch725680 (**52**), were obtained from the culture of a mangrove sediment-derived fungus *Penicillium pinophilum* SCAU037. Compounds **50** to **52** significantly inhibited α-glucosidase, with IC_50_ values of 51.9, 78.4, and 33.8 µM, respectively [26]. Two new compounds, **53** and **54**, which have not yet been named, along with two known compounds, peniciaculin A (**55**) and expansol D (**56**), were isolated from the culture of the endophytic fungus *Aspergillus flavus* QQSG-3 sourced from a fresh branch of Kandelia obobata collected in Huizhou, Guangdong, China. Compounds **53** to **56** strongly inhibited α-glucosidase, with IC_50_ values ranging from 1.5 to 4.5 µM [27]. The extraction and isolation of marine sediment *Penicillium species* collected in Vietnam yielded a known compound, 2′,3′-dihydrosorbicillin (**57**), which potentially inhibited α-glucosidase at a concentration of 2.0 mM, with an inhibition rate of 66.31% [28]. A new flavone, aspergivone B (**58**), was isolated from the fungus *Aspergillus candidus* cultured from the gorgonian coral Anthogorgia ochracea collected in the South China Sea. It slightly inhibited α-glucosidase, with an IC_50_ value of 244 µg/mL [29]. A new naphthoquinone, 5-hydroxy-2-methoxynaphtho[9–*c*]furan-1,4-dione (**59**), and a known compound, 2-acetyl-7-methoxybenzofuran (**60**), were obtained from the EtOAc extract of the mangrove-derived fungus *Daldinia eschscholtzii* HJ004. They potently inhibited α-glucosidase, with IC_50_ values of 5.7 and 1.1 µg/mL, respectively [30]. A chemical investigation of *P. brefeldianum* F4a, using an activity-guided isolation approach, led to the discovery of a novel compound, peniorcinol C (**61**). This compound significantly inhibited α-glucosidase, with an IC_50_ value of 38.98 µg/mL [31]. Two new de-*O*-methyllasiodiplodins, (3*R*,7*R*)-7-hydroxy-de-*O*-methyllasiodiplodin (**62**) and (3*R*)-5-oxo-de-*O*-methyllasiodiplodin (**63**), were isolated from the co-cultivation of the mangrove endophytic fungus *Trichoderma* sp. 307 and the aquatic pathogenic bacterium *Acinetobacter johnsonii* B2. Both **62** and **63** potently inhibited α-glucosidase, with IC_50_ values of 25.8 and 54.6 µM, respectively, surpassing the positive control acarbose (IC_50_ = 703.8 µM) [32]. Four new compounds, including two chlorinated diphenyl ethers, chrysine B (**64**) and chrysine C (**65**), one dichlorinated xanthone, chrysoxanthone (**66**), and a new compound dichloroorcinol (**67**), along with five known compounds, methyl 3′methoxy-3,5-dichloroasterric acid (**68**), 2,4-dichloroasterric acid (**69**), methyl chloroasterrate(**70**), mono-chlorosulochrin (**71**), and (+)-geodin (**72**), were isolated from a deep-sea-derived fungus *Penicillium chrysogenum* SCSIO 41001. Compounds **64**–**72** inhibited α-glucosidase, with IC_50_ values ranging from 0.04 to 0.35 mM (IC_50_ 0.28 mM for acarbose) [33]. A new phenylpropanoid derivative (**73**) was isolated from the liquid substrate fermentation cultures of the mangrove endopytic fungus *Aspergillus* sp. ZJ-68. It potently inhibited α-glucosidase, with an IC_50_ value of 12.4 µM [34]. A new furanone derivative, butanolide A (**74**), was isolated from the Antarctic marine-derived fungus *Penicillium* sp. S-1–18. It showed moderate PTP1B inhibitory activity, with an IC_50_ value of 27.4 μM [35]. Three known secondary metabolites, funalenone (**75**), aurasperone F (**76**), and fonsecin (**77**), were isolated from a marine-derived fungal strain *Aspergillus* sp. SF5929. These compounds inhibited PTP1B, with IC_50_ values ranging from 3.3 to 7.9 mM [36]. A new polyhydroxy p-terphenyl, asperterphenyllin A (**78**), was isolated from an endophytic fungus *Aspergillus candidus* LDJ-5. This compound inhibited PTP1B, with an IC_50_ value of 21µM [37].

The fermented extract of marine-derived fungal strain *Penicillium* spp. and *Eurotium* sp. yielded a compound, flavoglaucin (**79**), which inhibited PTP1B activity, with an IC_50_ value of 13.4 µM [38]. A bioassay-guided investigation of the methylethylketone extract of the marine-derived fungus *Penicillium* sp. JF-55 cultures led to the isolation of a new PTP1B-inhibitory styrylpyrone-type metabolite, penstyrylpyrone (**80**), and a known metabolite, anhydrofulvic acid (**81**). Both compounds inhibited PTP1B activity in a dose-dependent manner [39]. A chemical investigation of the marine-derived fungal strain *Penicillium glabrum* SF-7123 revealed two known secondary fungal metabolites, myxotrichin C (**82**) and deoxyfunicone (**83**). Compound **82** inhibited PTP1B, with an IC_50_ value of 19.2 µM, and compound **83** inhibited PTP1B, with an IC_50_ value of 24.3 µM [40]. Investigation of a marine-derived fungus *Penicillium* sp. SF-6013 led to the discovery of two known analogs, tanzawaic acids A (**84**) and B (**85**), which significantly inhibited PTP1B activity, with the same IC_50_ value of 8.2 µM [41]. A known prenylated flavanone derivative (**86**) from the culture broth of an Indonesian marine sponge-derived *Cladosporium* sp. TPU1507 inhibited PTP1B, with an IC_50_ value of 11 μM [42]. During a bioassay-guided study on the EtOAc extract of a marine-derived fungus *Cosmospora* sp. SF-5060, aquastatin A (**87**) was isolated as a PTP1B-inhibitory component, with an IC_50_ value of 0.19 µM [43]. Two new 3,4,6-trisubstituted α-pyrone derivatives, chrysopyrones A and B (**88** and **89**), isolated from *Penicillium chrysogenum* SCSIO 07007, inhibited PTP1B, with IC_50_ values of 9.32 and 27.8 μg/mL, respectively [44]. A new γ-pyrone-containing polyketide, fusaresters B (**90**), isolated from a marine-derived fungus *Fusarium* sp. Hungcl, had a PTP1B inhibition rate of 56% at 40 μM [45]. Linoleic acid (**91**), identified from the marine fungus *Eutypella* sp. F0219, enhanced mitochondrial oxidation through the FABP4 axis [46].

**Figure 2:**
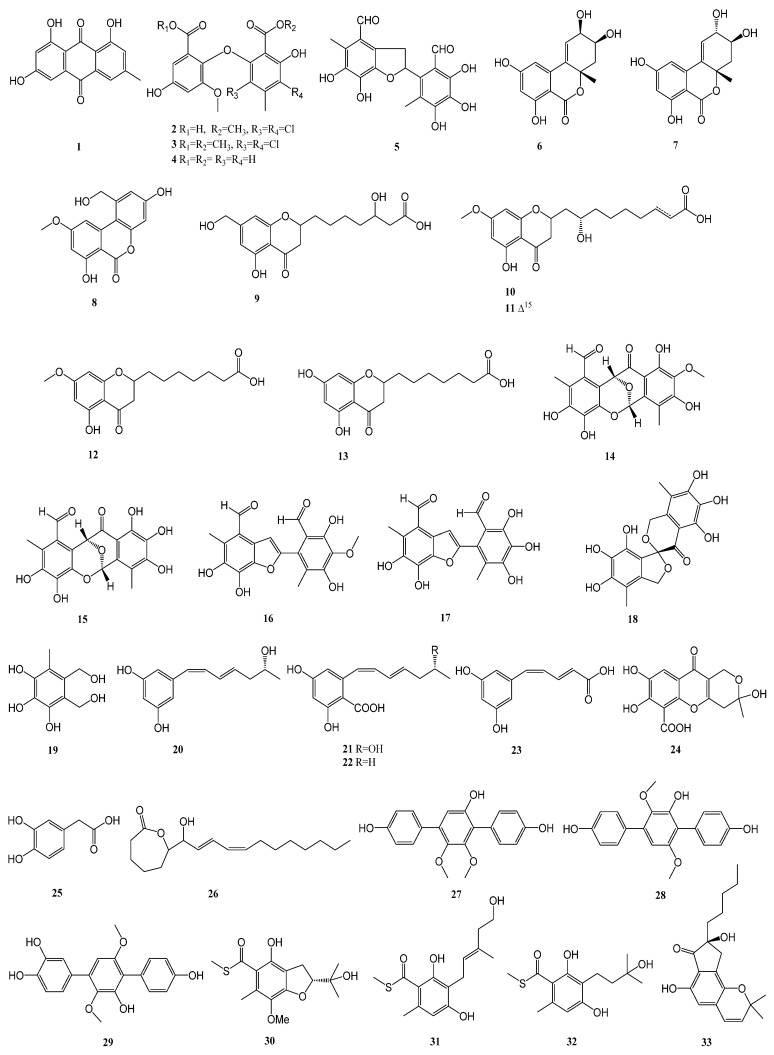

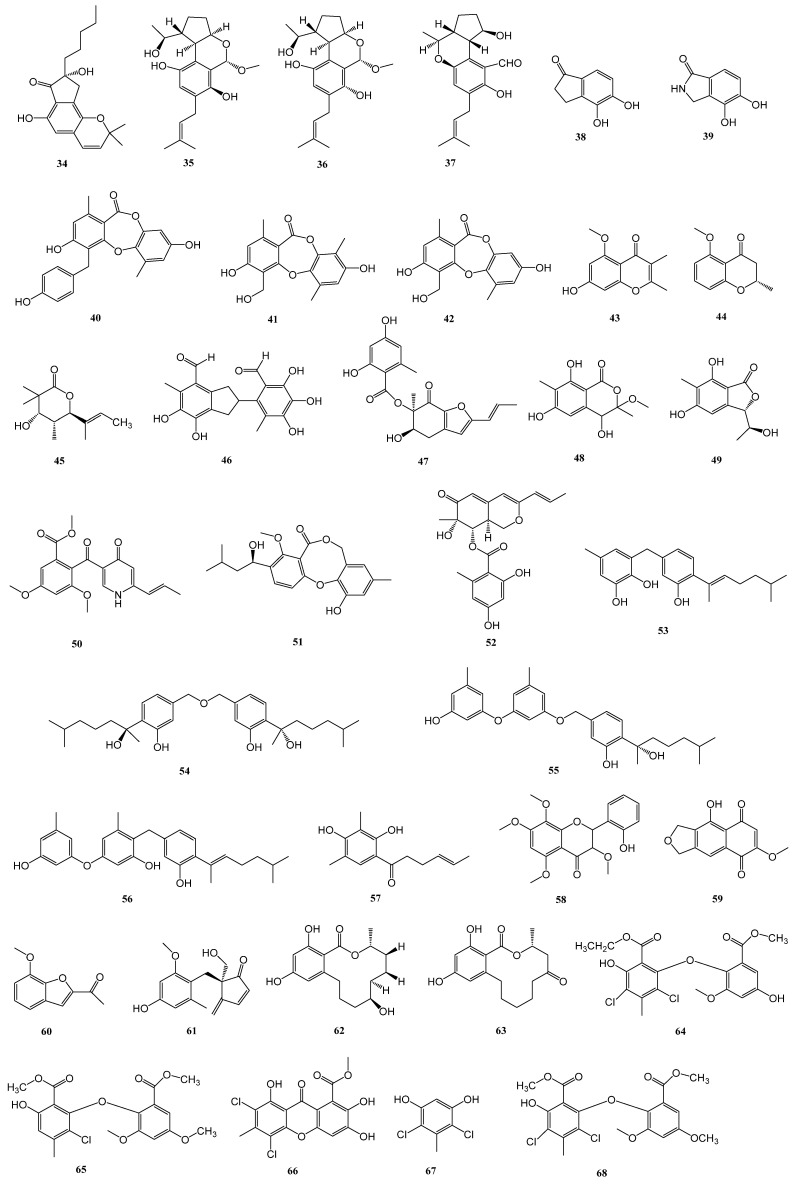

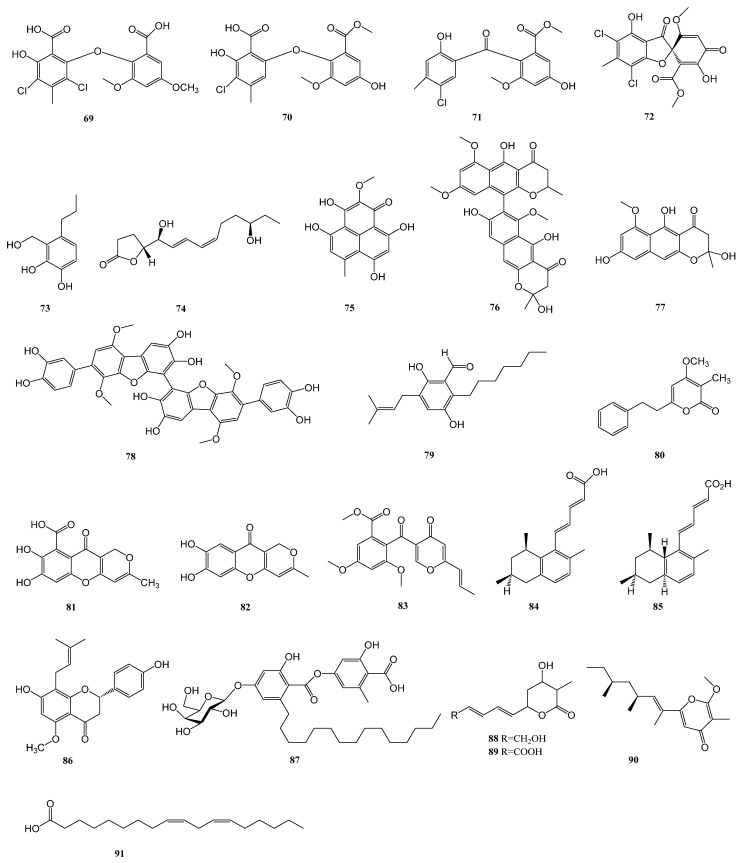
Structure diagram of polyketones.

Through statistical analysis, 91 polyketones were successfully isolated from 36 strains of marine-derived fungi. These compounds were found to display biological activity against diabetes-related enzymes. The structure is presented in Figure 2 below.

### 2.2. Alkaloid

Alkaloids, nitrogen-containing organic compounds, are ubiquitously present in nature. They are predominantly found in plants but also exist in animals and microorganisms. Their intricate structures, typically featuring nitrogen-atom-based rings, give rise to a wide variety of types [46]. Alkaloids possess significant biological activities and medicinal value. For instance, morphine is used for pain relief, quinine for malaria treatment, and caffeine stimulates the nervous system [47]. In agriculture, some alkaloids function as natural insecticides. Due to their unique properties, alkaloids are a focal point of research in drug development and natural product chemistry. Scientists explore biological resources to discover new alkaloids and study their mechanisms, aiming to develop better products for human health and agriculture.

Thiolactone (**92**) was isolated from *Meira* sp. 1210CH-42 and it inhibited α-glucosidase, with an IC_50_ value of 148.4 μM, outperforming acarbose (IC_50_ = 418.9 μM) [48]. A new tyrosine-derived metabolite, aspergillusol A (**93**), was isolated on a gram scale from the marine-derived fungus *Aspergillus aculeatus* CRI323-04. It selectively inhibited α-glucosidase from Saccharomyces cerevisiae but had no effect on the bacterial α-glucosidase from *Bacillus stearothermophilus* [49]. By feeding tryptophan to the marine-derived fungus *Aspergillus* sp. HNMF114 sourced from the bivalve mollusk Sanguinolaria chinensis, two known quinazoline-containing indole alkaloids, lapatin A (**94**) and scequinadoline E (**95**), were obtained. Both compounds inhibited α-glucosidase, with IC_50_ values of 7.18 and 5.29 mM, respectively [50]. Two new sambutoxin derivatives, sambutoxin A (**96**) and sambutoxin B (**97**), along with three known sambutoxin derivatives, (–)-sambutoxin (**98**), ilicicolin H (**99**), and deoxyleporin B (**100**), were isolated from the semimangrove endophytic fungus *Talaromyces* sp. CY-3. These compounds inhibited α-glucosidase, with IC_50_ values ranging from 12.6 ± 0.9 to 57.3 ± 1.3 μM, outperforming the positive control 1-deoxynojirimycin (IC_50_ = 80.8 ± 0.3 μM). Molecular docking analysis indicated that the activity potencies of compounds **96**–**98** correlated with the number of hydrogen bonds formed with α-glucosidase [51]. A new lumazine peptide, penilumamide K (**101**), was isolated from the deep-sea-derived fungus *Aspergillus* sp. SCSIO 41029. It showed significant inhibitory activity against α-glucosidase, with an IC_50_ value of 18.61 μΜ [52]. A new indole-diterpenoid, penpaxilloids A (**102**), along with the known compound paspalinine-13-ene (**103**), was isolated from the marine-derived fungus *Penicillium* sp. ZYX-Z-143. Compound **102** inhibited protein tyrosine phosphatase 1B (PTP1B), with an IC_50_ value of 8.60 ± 0.53 μM, while compound **103** inhibited α-glucosidase, with an IC_50_ value of 19.96 ± 0.32 μM [53]. From a mangrove fungus *Mycosphaerella* sp. SYSU-DZG01, a new metabolite, asperchalasine I (**104**), and a known compound, asperchalasine A (**105**), were isolated. Both compounds inhibited α-glucosidase, with IC_50_ values of 17.1 and 15.7 μM, respectively [24]. Two undescribed 4-quinolone alkaloids, (±)-oxypenicinolines A (**106**–**107**), and a known analog quinolactacide (**108**), were isolated from the mangrove-derived fungus *Penicillium steckii* SCSIO 41025. (±)-Oxypenicinoline A (**106**–**107**) and quinolactacide (**108**) inhibited α-glucosidase, with IC_50_ values of 317.8 and 365.9 μΜ, respectively, which were more potent than acarbose (461.0 μM) [54].

Chemical investigation of *P. brefeldianum* F4a led to the discovery of two known compounds, riboflavin (**109**) and indole-3-acetic acid (**110**). Compounds **109** and **110** showed PTP1B inhibitory activity, with IC_50_ values of 8.87 and 11.68 μM, respectively, and compound **110** also inhibited α-glycosidase, with an IC_50_ value of 21.48 μM [31]. Two new compounds, epipaxilline (**111**) and penerpene J (**112**), were isolated from the marine-derived fungus *Penicillium* sp. KFD28. They inhibited PTP1B, with IC_50_ values of 31.5 and 9.5 μM, respectively [55]. Three new indole-diterpenoids, penerpene O (**113**), penerpene P (**114**), and penerpene U (**115**), and a known analog, dehydroxypaxilline (**116**), were isolated from the marine soft coral-derived fungus *Aspergillus* sp. ZF-104. Compounds **113**–**116** inhibited PTP1B, with IC_50_ values ranging from 14.3 to 28.1 μM, comparable to that of the positive control NaVO₃ (IC_50_ = 33.6 μM) [56]. A known compound, emethacin C (**117**), was obtained from the marine-derived fungus *Aspergillus terreus* RA2905. It inhibited PTP1B, with an IC_50_ value of 12.25 μM [57]. Malformin A1 (**118**), a known secondary metabolite, was isolated from a marine-derived fungal strain *Aspergillus* sp. SF5929. It inhibited PTP1B, with an IC_50_ value of 5.2 ± 0.5 μM [36]. Bioassay-guided investigation of organic extracts from several marine-derived fungal species led to the isolation of fructigenine A (**119**), cyclopenol (**120**), echinulin (**121**), and viridicatol (**122**). These compounds inhibited PTP1B, with IC_50_ values of 10.7, 30.0, 29.4, and 64.0 μM, respectively [38]. Cladosporamide A (**123**) isolated from the culture broth of an Indonesian marine sponge-derived *Cladosporium* sp. TPU1507 inhibited PTP1B, with an IC_50_ value of 48 μM [42]. Penicopeptide A (**124**) was isolated from the deep-sea-derived *Penicillium solitum* MCCC 3A00215. It binds directly to GSK-3β (K_D_ = 177 nM), activating its phosphorylation and leading to β-catenin accumulation [58]. A new disubstituted maleimide, aspergteroid G (**125**), was isolated from the fermentation extract of the soft-coral-associated symbiotic and epiphytic fungus *Aspergillus terreus* EGF7-0-1. It is suppressed by glycogen synthase kinase-3 beta (GSK-3β) [59]. A new isopyrrolonaphthoquinone (**126**) was isolated from the fungal *Biscogniauxia mediterranea* LF657, which was from the Herodotes Deep (2800 m depth) in the Mediterranean Sea. It inhibited glycogen synthase kinase (GSK-3β), with an IC_50_ value of 8.04 μM [60]. A new fumiquinazoline alkaloid, scequinadoline D (**127**), was isolated and characterized from the marine fungus *Scedosporium apiospermum* F41-1. It acts through activation of the PPARγ pathway, stimulating the mRNA expression of FABP4 [61].

**Figure 3:**
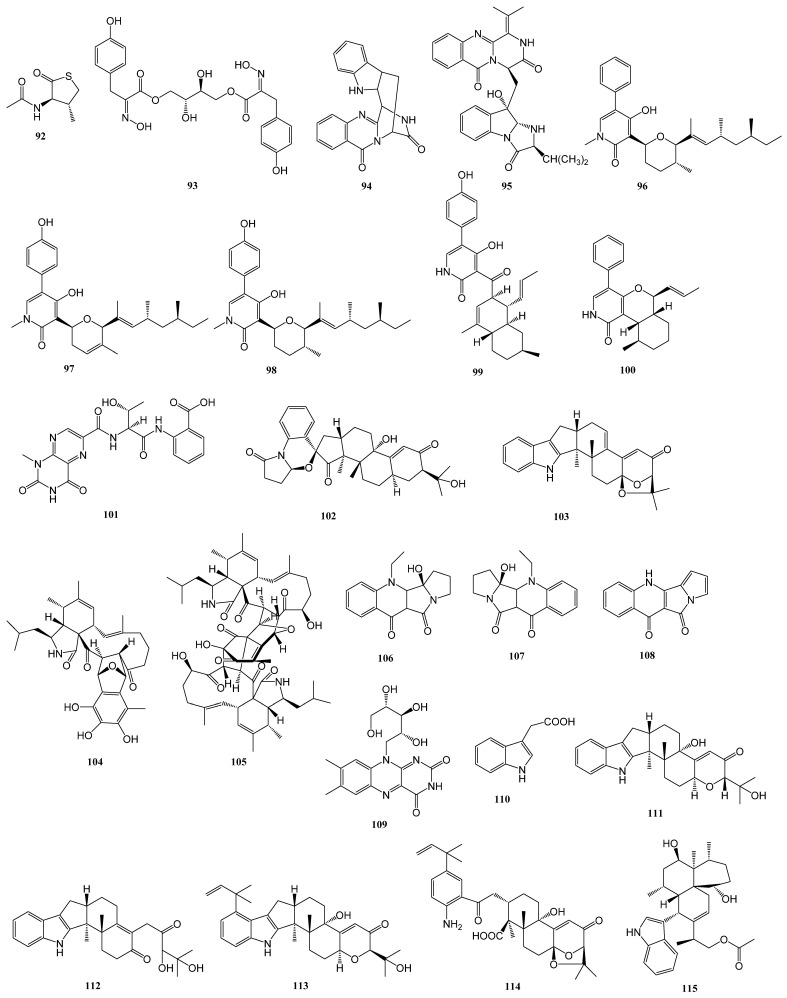

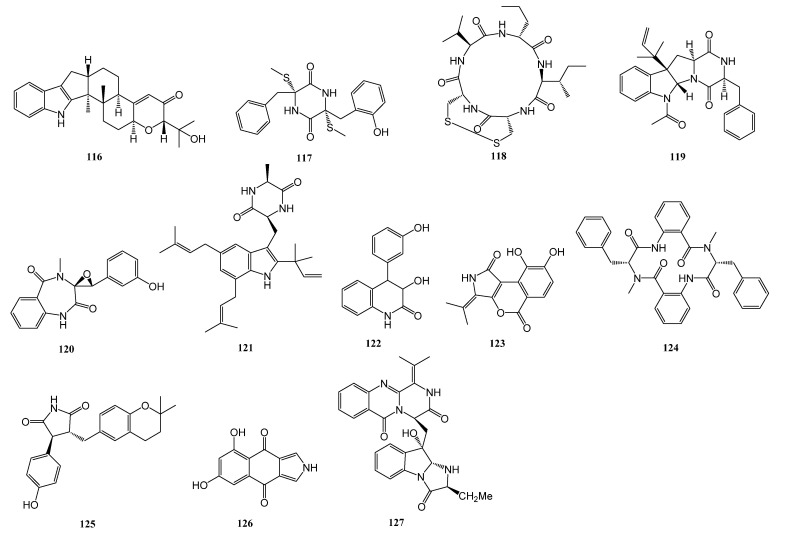
Alkaloid structure diagram.

Upon statistical analysis, 36 alkaloids were successfully isolated from 15 strains of marine-derived fungi. These compounds demonstrated biological activity against diabetes-related enzymes. The structure is presented in Figure 3 below.

### 2.3. Terpenoids

Terpenoids, which are natural organic compounds widely distributed in nature, are composed of linked isoprene units that form a distinctive molecular framework, contributing to their rich structural diversity. Based on the number of isoprene units, they can be classified into monoterpenes, sesquiterpenes, diterpenes, and so on. These compounds mainly originate from plants, and some are also synthesized by microorganisms and marine organisms. Terpenoids have diverse bio-activities, such as anti-inflammatory, antibacterial, and anti-tumor effects [62]. Notably, they hold promise in diabetes prevention and treatment by regulating insulin secretion and improving insulin resistance. They also find applications in the spice industry and agriculture. Given their unique features and broad prospects, terpenoids are a research focus in multiple fields and are expected to contribute to diabetes drug R&D in the future.

Three new meroterpenoids, chrodrimanin O (**128**), chrodrimanin R (**129**), and chrodrimanin S (**130**), along with a known compound **131**, were isolated from the fermentation broth of *Penicillium* sp. SCS-KFD09 sourced from the marine worm Sipunculus nudus collected in Haikou Bay, China. These compounds inhibited protein tyrosine phosphatase 1B (PTP1B), with IC_50_ values of 71.6, 62.5, 63.1, and 39.6 μM, respectively [63]. A new ∆^8,9^-steroid (**132**) and a known analog (**133**) were isolated from *Meira* sp. 1210CH-42. They inhibited α-glucosidase, with IC_50_ values of 86.0 and 279.7 μM, respectively, outperforming acarbose (IC_50_ = 418.9 μM) [48]. Chemical analysis of the EtOAc extract from the fermentation broth of the marine-derived fungus *Trametes* sp. ZYX-Z-16 led to the identification of two ergostane steroid analogs: ergosta-4,6,8,22*E*-tetraene-3-one (**134**) and 14α-hydroxyergosta-4,7,22*E*-triene-3,6-dione (**135**). These compounds inhibited the enzyme, with IC_50_ values of 104.1 and 111.3 μM, respectively, compared to the positive control acarbose (304.6 μM) [64]. A novel drimane sesquiterpene (**136**) was isolated from the marine-derived fungus *Penicillium* sp. TW58-16, showing strong α-glucosidase inhibitory effects, with inhibition rates of 35.4% at 400 μM [17]. Chemical investigation of the culture broth of *P. levitum* strain N33.2, extracted with ethyl acetate, led to the isolation of three ergostane-type steroid components: ergosterol peroxide (**137**) and (3β,5α,22*E*)-ergosta-6,8,22-triene-3,5-diol (**138**). Compounds **137** and **138** inhibited α-glucosidase, with IC_50_ values of 21.89 (51.14 μM equivalent) and 25.81 μg/mL (60.02 μM equivalent), respectively, more potently than acarbose (IC_50_ = 235.56 μg/mL) [65]. Demethylincisterol A2 (**139**), a known compound, was isolated from the soft-coral-derived fungus *Aspergillus hiratsukae* SCSIO 5Bn1003. It inhibited α-glucosidase, with an IC_50_ value of 35.73 μM, close to acarbose (IC_50_ = 32.92 μM) [66]. Study of the ethyl acetate extract from the deep-sea-derived fungus *Penicillium thomii* YPGA3 resulted in the isolation of known analogs austalide P (**140**) and a derivative agathic acid (**141**). Compounds **140** and **141** inhibited α-glucosidase, with IC_50_ values of 910 and 525 μM, respectively, more actively than the positive control acarbose (1.33 μM) [67].

GKK1032B (**142**), isolated from the marine-derived fungal strain *Penicillium* sp. SCSIO 41512, inhibited protein tyrosine phosphatases PTP1B, with an IC_50_ value of 25 μM [68]. Four known analogs, ascomylactam B (**143**) and phomapyrrolidone A-C (**144**–**146**), were isolated from the marine-derived fungus *Microascus* sp. SCSIO 41821. These compounds inhibited PTP1B, with IC_50_ values ranging from 8.7 to 11 μM [69]. A new compound **147** from the marine sponge-derived fungus *Penicillium chrysogenum* exhibited moderate activity against PTP1B at a concentration of 30 μM [70].

Chemical investigation of the marine-derived fungal isolate *Penicillium* sp. SF-5497 led to the isolation of a new preaustinoid-related meroterpenoid, preaustinoid A7 (**148**), along with a known metabolite (**149**). Both compounds inhibited PTP1B activity, with IC_50_ values of 17.6 and 58.4 μM, respectively, and compound **148** inhibited PTP1B in a noncompetitive manner [71]. Three novel indole-terpenoids, penerpene E (**150**), penerpene F (**151**), and penerpene H (**152**), along with a known compound 7-hydroxypaxilline-13-ene (**153**), were isolated from the marine-derived fungus *Penicillium* sp. KFD28. These compounds inhibited PTP1B, with IC_50_ values ranging from 13 to 27 μM [72]. Two unusual indole-terpenoids, penerpene A (**154**) and penerpene B (**155**), isolated from the same fungus, inhibited PTP1B with IC_50_ values of 1.7 and 2.4 μM, respectively [73]. A new merosesquiterpene, verruculide A (**156**), along with known congeners chrodrimanin A (**157**) and chrodrimanin H (**158**), was isolated from the culture broth of Indonesian ascidian-derived *Penicillium verruculosum* TPU1311. These compounds inhibited PTP1B, with IC_50_ values of 8.4, 8.5, and 14.9 μM, respectively [74].

**Figure 4:**
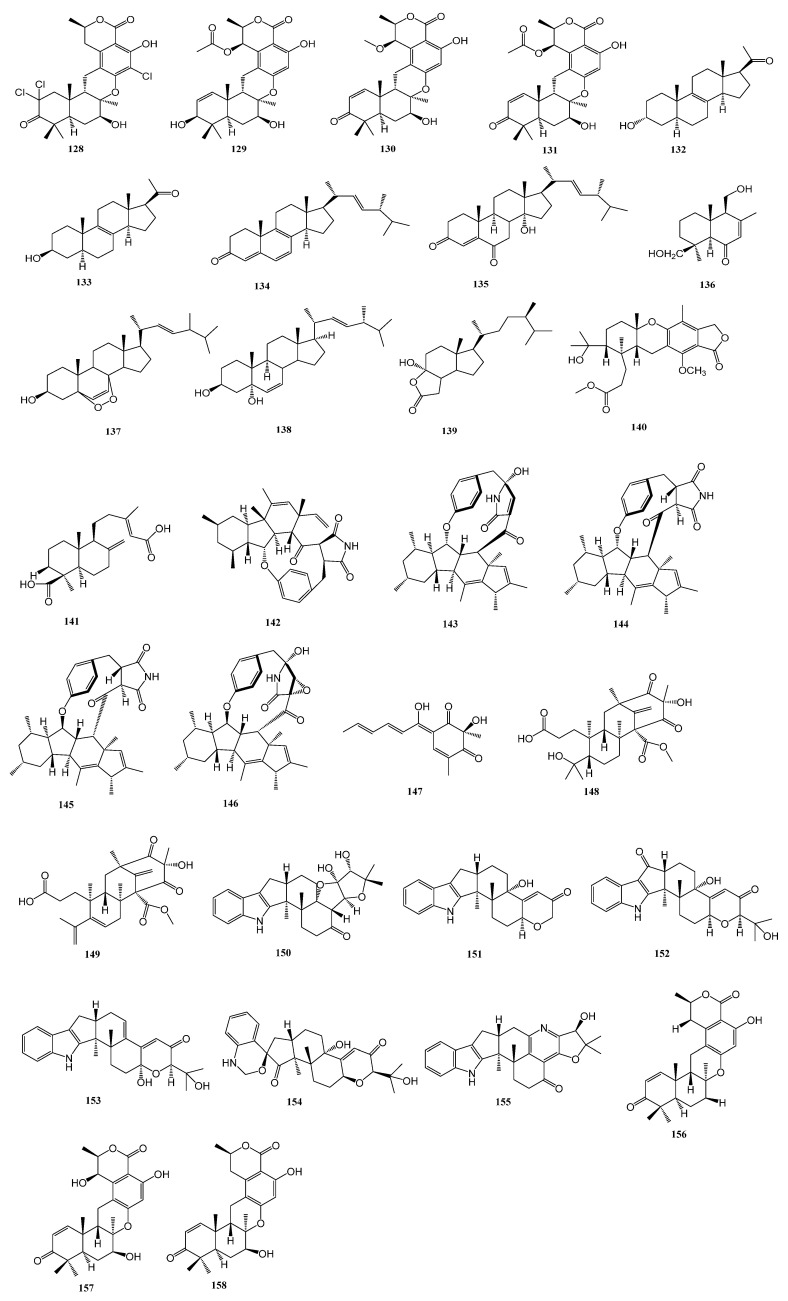
Terpenoids diagram of terpenoids.

Upon statistical analysis, 31 terpenoids were successfully isolated from 12 strains of marine-derived fungi. These compounds possess biological activity against diabetes-related enzymes. The structure is presented in Figure 4 below.

### 2.4. Lignan

Lignans, a class of natural organic compounds, are formed when two phenylpropanoid derivatives link via the β-carbon atoms of their side-chains. They have unique and diverse structures and are widely found in plants such as Schisandra chinensis and Forsythia suspensa [75]. Lignans exhibit diverse biological activities, particularly attracting attention in diabetes research. Some can inhibit α-glucosidase, while others enhance insulin sensitivity by regulating the insulin signaling pathway, and their antioxidant and anti-inflammatory properties are beneficial for countering diabetes-related oxidative stress and inflammation, holding promise for diabetes prevention and treatment.

Three new butenolide derivatives, flavipesolides A-C (**159**–**161**), were isolated alongside two known compounds, 5-[(3,4-dihydro-2,2-dimethyl-2*H*-1-benzopyran-6-yl)methyl]-3-hydroxy-4-(4-hydroxyphenyl)-2(5*H*)furanone (**162**) and aspernolide A (**163**), from the marine-derived *Aspergillus flavipes* HN4-13 obtained from a Lianyungang coastal sediment sample. Compounds **159** to **161** inhibited α-glucosidase, with K_i_/IC_50_ values of (2.5, 19)/44, (3.4, 14)/57, and (9.2, 4.7)/95 μM, respectively. Compounds **162** and **163** inhibited α-glucosidase non-competitively, with K_i_/IC_50_ values of 0.43/34 and 2.1/37 μM, respectively. Compared to compound **161**, esterified compounds **159** and **160** exhibited stronger α-glucosidase inhibitory activity [13]. Three new butenolide derivatives, (±)-asperteretal D (**164**–**165**) and asperteretal E (**166**), with a rare 2-benzyl-3-phenyl substituted lactone core, along with four known analogs, flavipesolide B (**167**), flavipesolide C (**168**), butyrolactone I (**169**), and 5-[(3,4-dihydro-2,2-dimethyl-2*H*-1-benzopyran-6-yl)methyl]-3-hydroxy-4-(4-hydroxyphenyl)-2(5*H*)-furanone (**170**), were derived from *Aspergillus terreus* from the marine sponge Phakellia fusca. Compounds **164** to **170** potently inhibited α-glucosidase, with IC_50_ values ranging from 8.65 to 20.3 mM [76]. A new butenolide derivative, versicolactone G (**171**), was isolated from a coral-associated *Aspergillus terreus*. It inhibited α-glucosidase with a potent IC_50_ value of 104.8 ± 9.5 μM, lower than that of the positive control acarbose [77]. Four known compounds, butyroscavin (**172**), butyrolactone II (**173**), aspernolide D (**174**), and aspulvinone E (**175**), were obtained from the deep-sea-derived fungus *Aspergillus* sp. SCSIO 41029. They had significant α-glucosidase inhibitory potency, with IC_50_ values ranging from 18.61 to 109.06 μM [52].

Three known analogs, butyrolactone VII (**176**), aspernolide A (**177**), and aspernolide E (**178**), were isolated from the ethyl acetate extract of the deep-sea-derived *Aspergillus terreus* YPGA10. These compounds inhibited α-glucosidase, with IC_50_ values of 1.37, 6.98, and 8.06 μM respectively, much lower than that of the positive control acarbose [78]. Two known compounds, (+)-3′,3′-di-(dimethylallyl)-butyrolactone II (**179**) and 3-hydroxy-5-(4-hydroxybenzyl)-4-(4-hydroxyphenyl)furan-2(5*H*)-one (**180**), were isolated from *Aspergillus terreus* SCAU011 in the rhizosphere sediment of the mangrove plant Rhizophora stylosa. Compounds **179** and **180** inhibited α-glucosidase, with IC_50_ values of 56.1 and 12.9 μM, respectively [79]. A racemate of a novel diphenolic derivative, (±)-tylopilusin D (**181**), was isolated from the marine-derived fungal strain *Aspergillus* sp. SF5929. It inhibited PTP1B activity, with an IC_50_ value ranging from 8.1 ± 0.4 mM [36].

**Figure 5:**
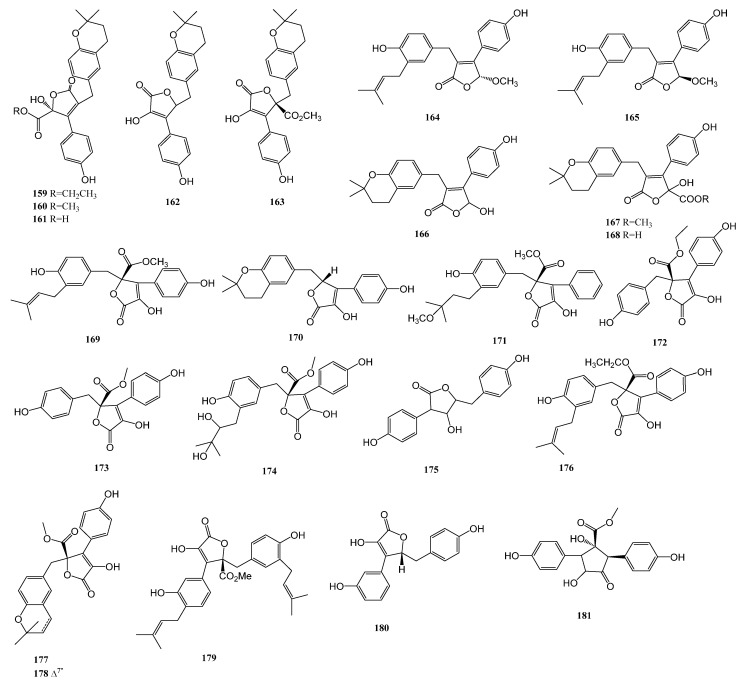
Lignan diagram of lignans.

After statistical analysis, 23 lignans were successfully isolated from six strains of marine-derived fungi. These compounds were determined to possess biological activity against diabetes-related enzymes. The structure is presented in Figure 5 below.

**Table 1:** Enzyme activity of compounds associated with diabetes.

Compound Number	Derived Fungi	Sampling Source	Activity-Related Enzyme	Reference
**1**–**5**	*Aspergillus flavipes* HN4-13	Lianyungang coastal sediment sample	α-glucosidase	[13]
**159**–**163**				
**6**–**8**	*Alternaria alternata* LW37	a deep-sea sediment sample collected at a depth of 2623 m in the Southwest Indian Ridge	α-glucosidase	[14]
**9**–**13**	*Penicillium thomii YPGA3*	the sediments of the Yap Trench	α-glucosidase	[15]
**14**–**19**	*Aspergillus insulicola*	deep-sea sediments, which were collected from the South China Sea at a depth of 2500 m.	α-glucosidase	[16]
**20**–**26**	*Penicillium* sp. TW58-16	hydrothermal vent sediment, collected from Kueishantao, Taiwan	α-glucosidase	[17]
**136**				
**27**–**29**	*Aspergillus candidus* HM5-4	sponges from the South China Sea	α-glucosidase	[18]
**30**–**32**	*Talaromyces indigoticus* FS688	the South China Sea (118°19.692′ N, 20°38.982′ E; depth 2372 m)	α-glucosidase	[19]
**33**–**37**	*Eurotium* sp. SCSIO F452	sediment samples from northern South China Sea	α-glucosidase	[20]
**38**–**39**	*Meira* sp. 1210CH-42	a seawater sample collected at the Chuuk Islands, Federated States of Micronesia	α-glucosidase	[21]
**40**–**42**	*Trichoderma* sp. 307	the stem bark of Clerodendrum inerme, which was collected from Zhanjiang Mangrove National Nature Reserve in Guangdong Province, China.	α-glucosidase	[22]
**43**–**45**	*Daldinia eschscholtzii* HJ004	the mangrove Bruguiera sexangula var. Rhynchopetala collected in the South China Sea	α-glucosidase	[23]
**46**	*Mycosphaerella* sp. SYSU-DZG01	the fruit of the marine mangrove plant Bruguiera collected in 2014 in Hainan Dongzhai Harbor Mangrove Reserve	α-glucosidase	[24]
**104**–**105**				
**47**–**49**	*Aspergillus* sp. 16-5B	the leaves of Sonneratia apetala, which was collected from Dongzhaigang Mangrove National Nature Reserve in Hainan Island, China	α-glucosidase	[25]
**50**–**52**	*Penicillium pinophilum* SCAU037	the roots of a mangrove plant Rhizophora stylosa on the Techeng Isle, China	α-glucosidase	[26]
**53**–**56**	*Aspergillus flavus* QQSG-3	a fresh branch of Kandelia obobata, which was collected from Huizhou city in the province of Guangdong, China	α-glucosidase	[27]
**57**	*Penicillium strain* M30	the sediment that was collected at a depth of 14 m sea at the Co To island, Northern Vietnam	α-glucosidase	[28]
**58**	*Aspergillus candidus*	the gorgonian coral Anthogorgia ochracea collected from the South China Sea	α-glucosidase	[29]
**59**–**60**	*Daldinia eschscholtzii* HJ004	the stem of mangrove Brguiera sexangula var. rhynchopetala, collected in the South China Sea	α-glucosidase	[30]
**61**	*Penicillium brefeldianum* F4a	the roots of H. cordata	α-glucosidase and PTP1B	[31]
**109**			α-glucosidase	
**110**			α-glucosidase and PTP1B	
**62**–**63**	*Trichoderma* sp. 307	the stem bark of Clerodendrum inerme, collected in Zhanjiang Mangrove National Nature Reserve in Guangdong Province, China	α-glucosidase	[32]
**64**–**72**	*Penicillium chrysogenum* SCSIO 41001	the deep sea sediment of Indian Ocean (Lat: 10.00371667° N, long: 88.72803333° E) at a depth of 3386 m	α-glucosidase	[33]
**73**	*Aspergillus* sp. ZJ-68	fresh leaves of the mangrove plant Kandelia candel, which were collected from the Zhanjiang Mangrove Nature Reserve in Guangdong Province, China	α-glucosidase	[34]
**74**	*Penicillium* sp. S-1-18	the Antarctic seabed sediments (47.09° W, 62.05° S, the depth of 1393 m)	PTP1B	[35]
**75**–**77**	*Aspergillus* sp. SF5929	marine-derived	PTP1B	[36]
**118**				
**181**				
**78**	*Aspergillus candidus* LDJ-5	the root of Rhizophora apiculata Blume in the Sanya Bailu Park of Hainan Province, China	PTP1B	[37]
**79**	*Penicillium* sp. SF-5203	an intertidal sediment sample collected from Wan Island, Korea	PTP1B	[38]
**119**–**122**				
**80**–**81**	*Penicillium* sp. JF-55	an unidentified sponge that was manually collected using scuba equipment off the shores of Jeju Island	PTP1B	[39]
**82**–**83**	*Penicillium glabrum* SF-7123	sediments that were collected using a dredge at the Ross Sea (77°34.397′ N, 166°10.865′ W)	PTP1B	[40]
**84**–**85**	*Penicillium* sp. SF-6013	the sea urchin Brisaster latifrons collected from the Sea of Okhotsk (N 53°22.626′ E 144°24.200′)	PTP1B	[41]
**86**	*Cladosporium* sp. TPU1507	an unidentified marine sponge collected at Manado, Indonesia	PTP1B	[42]
**123**				
**87**	*Cosmospora* sp. SF-5060	an inter-tidal sediment collected at Gejae Island	PTP1B	[43]
**88**–**89**	*Penicillium chrysogenum* SCSIO 07007	deep-sea hydrothermal vent environment sample collected from the Western Atlantic	PTP1B	[44]
**90**	*Fusarium* sp. Hungcl	the soil, collected from the Futian Mangrove Reserve in Shenzhen, Guangdong Province	PTP1B	[45]
**91**	*Eutypella* sp. F0219	a marine sediment sample collected in the northern part of the South China Sea (GPS 114.6609° E, 21.5942° N) at a water depth of 75 m	FABP4	[46]
**92**	*Meira* sp. 1210CH-42	a seawater sample collected at Chuuk Islands, Federated States of Micronesia	α-glucosidase	[48]
**132**–**133**				
**93**	*Aspergillus aculeatus* CRI323-04	the marine sponge Xestospongia testudinaria (specimen no. CRI323)	α-glucosidase	[49]
**94**–**95**	*Aspergillus* sp. HNMF114	the bivalve mollusk Sanguinolaria chinensis	α-glucosidase	[50]
**96**–**100**	*Talaromyces* sp. CY-3	the fresh leaves of the semimangrove Hibiscus tiliaceus	α-glucosidase	[51]
**101**	*Aspergillus* sp. SCSIO 41029	a deep-sea sediment sample of South China Sea	α-glucosidase	[52]
**172**–**175**				
**102**	*Penicillium* sp. ZYX-Z-143	an arthropod, Dardanus scutellatus, collected from Yinyu Island, one of the Paracel Islands in South China Sea, Hainan province (16°35′03″ N, 111°42′39″ E)	PTP1B	[53]
**103**			α-glucosidase	
**106**–**108**	*Penicillium steckii* SCSIO 41025	the root of A. marina (Forsk.) Vierh. (Acanthaceae) collected from the mangrove wetland in Zhanjiang, Guangdong province, China (coordinates 21.235° N, 110.451° E)	α-glucosidase	[54]
**111**–**112**	*Penicillium* sp. KFD28	Meretrix lusoria, collected from Haikou Bay, China	PTP1B	[55]
**113**–**116**	*Aspergillus* sp. ZF-104	a marine soft coral in Haikou Bay, Hainan province, China	PTP1B	[56]
**117**	*Aspergillus* terreus RA2905	a piece of fresh tissue from the inner part of the sea hare Aplysia pulmonica, collected from the Weizhou coral reefs in the South China Sea	PTP1B	[57]
**124**	*Penicillium solitum* MCCC 3A00215	deep-sea-derived	GSK-3β	[58]
**125**	*Aspergillus terreus* EGF7-0-1	soft coral in the South China Sea	GSK-3β	[59]
**126**	*Biscogniauxia*	the sediment of the Herodotes Basin (2800 m water depth)	GSK-3β	[60]
**127**	*Scedosporium apiospermum* F41-1	the inner tissue of the soft coral Lobophytum crassum collected from Hainan Sanya National Coral Reef Reserve, People’s Republic of China	FABP4	[61]
**128**–**131**	*Penicillium* sp. SCS-KFD09	a marine worm, Sipunculusnudus, from Haikou Bay, China	PTP1B	[63]
**134**–**135**	*Trametes* sp. ZYX-Z-16	an unidentified sea snail collected from Silver Island, Xisha, South Sea, China	α-glucosidase	[64]
**137**–**138**	*Penicillium levitum* N33.2	the leaf of seagrass Enhalus acoroides (Hydrocharitaceae, Alismatales) obtained at Nhatrang bay, Khanhhoa, Vietnam (N 12°59.243′, E 109°21.965′) and symbolized as N33.2.	α-glucosidase	[65]
**139**	*Aspergillus hiratsukae* SCSIO 5Bn1003	a coral sample collected from the South China Sea	α-glucosidase	[65]
**140**–**141**	*Penicillium thomii* YPGA3	deep sea water at a depth of 4500 m in the Yap Trench (West Pacific Ocean)	α-glucosidase	[67]
**142**	*Penicillium* sp. SCSIO 41512	a soft coral of the South China Sea	PTP1B	[68]
**143**–**146**	*Microascus* sp. SCSIO 41821	a gorgonian Melitodes squamata collected from the South China Sea, Sanya (18°11′ N, 109°25′ E), Hainan, China	PTP1B	[69]
**147**	*Penicillium chrysogenum*	Plakortis simplex collected in Xisha islands, China	PTP1B	[70]
**148**–**149**	*Penicillium* sp. SF-5497	a sample of sea sand collected at Gijang-gun, Busan (35°22.2257′ N; 129°23.9238′ E)	PTP1B	[71]
**150**–**153**	*Penicillium* sp. KFD28	a bivalve mollusk, Meretrix lusoria, collected from Haikou Bay, China	PTP1B	[72]
**154**–**155**	*Penicillium* sp. KFD28	a bivalve mollusk, Meretrix lusoria, collected from Haikou Bay, China	PTP1B	[73]
**156**–**158**	*Penicillium verruculosum* TPU1311	an ascidian Polycarpa aurata collected in Indonesia	PTP1B	[74]
**164**–**170**	*Aspergillus terreus*	marine-derived	α-glucosidase	[76]
**171**	*Aspergillus terreus*	the coral Sarcophyton subviride, which was collected from the coast of Xisha Island in the South China Sea	α-glucosidase	[77]
**176**–**178**	*Aspergillus terreus* YPGA10	the deep-sea water at a depth of 4159 m in the Yap Trench (West Pacific Ocean)	α-glucosidase	[78]
**179**–**180**	*Aspergillus terreus* SCAU011	the rhizosphere sediment of a mangrove plant Rhizophora stylosa collected on the Techeng Isle, China	α-glucosidase	[79]

## 3. Discussion

Natural products, serving as a crucial reservoir of bioactive substances, harbor a vast array of active products with diverse functions. In our research on natural products derived from marine fungi, we zeroed in on their correlation with diabetes-related enzyme activity. A total of 181 natural products sourced from marine fungi and exhibiting diabetes-related enzyme activity were meticulously counted in this study, with the aim of delving deeper into their latent value in the realm of diabetes prevention and management.

**Figure 6:**
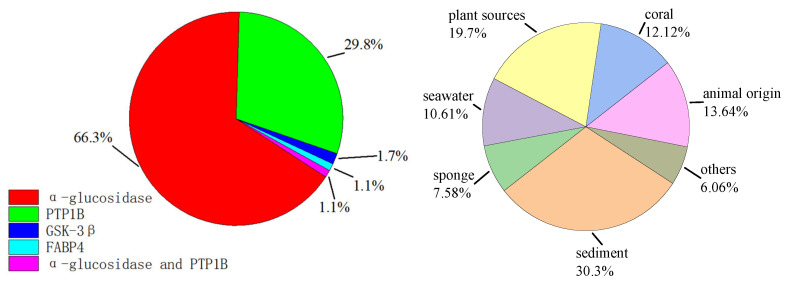
Percentage distributions of compounds against different diabetes-related enzymes (**left**) and strain sources (**right**).

The study findings revealed that among these 181 natural products, compounds associated with alpha-glycosidase constituted the largest proportion. This underscores the pivotal role these compounds play in modulating blood-sugar metabolism (Table 1). α-glycosidase inhibitors can effectively impede the digestion and absorption of carbohydrates, thus curbing the sharp increase in post-meal blood glucose levels. Compounds related to PTP1B ranked second. As a key negative regulator within the insulin signaling pathway, modulating the activity of PTP1B contributes to the alleviation of insulin resistance. Conversely, the quantity of compounds linked to GSK-3β and FABP4 was relatively scarce. Significantly, two compounds, **61** and **110**, were found to be associated with both α-glycosidase and PTP1B. This implies that these natural products might exert multiple mechanisms of action in regulating the activity of diabetes-related enzymes, thereby furnishing novel insights for the development of drugs for treating diabetes (Figure 6).

The chart illustrates a diverse range of strain sources. Sediment, accounting for 30.30%, provides a nutrient-rich and complex environment that may be conducive to the growth of fungi associated with diabetes-related enzyme-active secondary metabolites. Plant sources, representing 19.70% of the total, may foster the production of potentially valuable secondary metabolites by fungi due to plant–fungus symbiotic relationships. Seawater, making up 10.61%, has a unique environment that could enable fungi to develop distinctive metabolic pathways, leading to the generation of relevant bioactive substances (Figure 6). Animal sources, corals, and sponges, each with their respective proportions, may induce the synthesis of specific secondary metabolites in fungi due to their specialized ecological settings. The “others” category, constituting 6.06%, also holds exploration potential. These diverse sources offer abundant resources for the discovery of fungal secondary metabolites with diabetes-related enzyme activities.

Marine fungal natural products show great potential in diabetes-related enzyme activity research, yet drug development based on them is scarce. This is mainly due to the late start of research after 2000. Compared with traditional drug R&D, the data and theoretical basis are weak, impeding the transition from basic research to drug development. Also, their low yield and difficult extraction cannot meet the sample needs for in-depth research, blocking the analysis of the interaction mechanism between active ingredients and diabetes-related enzymes. However, there is hope for the future. As research advances, more data will support drug development. Moreover, advanced technologies like genetic engineering and synthetic biology may precisely regulate marine fungal metabolic pathways, increase yields, and promote diabetes treatment drug R&D, bringing more hope to patients.
